# Spatial architecture of regulatory T-cells correlates with disease progression in patients with nasopharyngeal cancer

**DOI:** 10.3389/fimmu.2022.1015283

**Published:** 2022-11-10

**Authors:** Fengge Zhou, Gulidanna Shayan, Shiran Sun, Xiaodong Huang, Xuesong Chen, Kai Wang, Yuan Qu, Runye Wu, Ye Zhang, Qingfeng Liu, Jianghu Zhang, Jingwei Luo, Xinqi Shi, Yang Liu, Bin Liang, Ye-Xiong Li, Jingbo Wang, Junlin Yi

**Affiliations:** ^1^ Department of Radiation Oncology, National Cancer Center/National Clinical Research Center for Cancer/Cancer Hospital, Chinese Academy of Medical Sciences and Peking Union Medical College, Beijing, China; ^2^ Tumor Research and Therapy Center, Shandong Provincial Hospital Affiliated to Shandong First Medical University, Jinan, Shandong, China; ^3^ Department of Radiation Oncology, National Cancer Center/National Clinical Research Center for Cancer/ Hebei Cancer Hospital, Chinese Academy of Medical Sciences, Langfang, China

**Keywords:** tumor infiltrating lymphocytes (TILs), programmed cell death ligand 1 (PDL1), nasopharyngeal carcinoma (NPC), cell spatial distribution, regulatory T cells (Tregs), cytotoxic T lymphocytes (CTLs), immune supression, proximity

## Abstract

**Purpose:**

This study aims to investigate the prognostic value of composition and spatial architecture of tumor-infiltrating lymphocytes (TILs) as well as PDL1 expression on TILs subpopulations in nasopharyngeal carcinoma (NPC).

**Methods:**

A total of 121 patients with NPC were included and divided into two groups: favorable (n = 68) and unfavorable (n = 53). The archived tumor tissues of the included patients were retrieved, and a tissue microarray was constructed. The density and spatial distribution of TILs infiltration were analyzed using the multiplex fluorescent immunohistochemistry staining for CD3, CD4, CD8, Foxp3, cytokeratin (CK), PDL1, and 4′,6-diamidino-2-phenylindole (DAPI). The infiltration density of TILs subpopulations and PDL1 expression were compared between the two groups. The Gcross function was calculated to quantify the relative proximity of any two types of cells. The Cox proportional hazards regression model was used to identify factors associated with overall survival (OS) and disease-free survival (DFS).

**Results:**

The densities of regulatory T-cells (Tregs), effector T-cells (Teffs), PDL1+ Tregs, and PDL1+ Teffs were significantly higher in patients with unfavorable outcomes. PDL1 expression on tumor cells (TCs) or overall TILs was not associated with survival. Multivariate analysis revealed that higher PDL1+ Tregs infiltration density was independently associated with inferior OS and DFS, whereas Tregs infiltration density was only a prognostic marker for DFS. Spatial analysis revealed that unfavorable group had significantly stronger Tregs and PDL1+ Tregs engagement in the proximity of TCs and cytotoxic T lymphocyte (CTLs). Gcross analysis further revealed that Tregs and PDL1+ Tregs were more likely to colocalize with CTLs. Moreover, increased G_TC_ : _Treg_ (Tregs engagement surrounding TCs) and G_CTL_ : _PDL1+ Treg_ were identified as independent factors correlated with poor outcomes.

**Conclusion:**

TILs have a diverse infiltrating pattern and spatial distribution in NPC. Increased infiltration of Tregs, particularly PDL1+ Tregs, as well as their proximity to TCs and CTLs, correlates with unfavorable outcomes, implying the significance of intercellular immune regulation in mediating disease progression.

## Background

Nasopharyngeal carcinoma (NPC) is characterized by its close association with Epstein–Barr virus (EBV) infection, poor differentiation, and sensitivity to radiotherapy and chemotherapy (1, 2). Intensity-modulated radiotherapy (IMRT) and advanced chemotherapeutic regimens have provided excellent overall loco-regional management for NPC (3–5). However, distant metastasis and local recurrence continue to occur in approximately 30%–40% of patients, and the response rate to immune checkpoint inhibitors is only 20%–30% (6–11). Therefore, it is crucial to identify additional robust prognostic markers of NPC and guide treatment beyond the well-known staging system and EBV DNA load (12).

The tumor microenvironment (TME) is an intricately organized landscape occupied by infiltrating immune cells, epithelial cells, vascular and lymphatic vessels, cytokines, and chemokines (13). The tumor immune microenvironment (TIME) is critical in the development and progression of many solid tumors (14–19). TIME analysis reveals the diverse composition and functional states of immune cells (20). Tumor-infiltrating lymphocytes (TILs), being the most important component of TIME, play a vital role in mediating antitumor immunity in the TME. Previous studies have demonstrated that TILs have a prognostic impact on a variety of solid cancers (15–19). Nevertheless, tumor cells (TCs) can evade immune surveillance in a variety of ways, including upregulating immune checkpoint receptor ligands such as programmed death-ligand 1 (PDL1) (21). Furthermore, regulatory T-cells (Tregs) and other suppressive signals can enhance tumor progression by attenuating antitumor immunity (13–15, 20).

A few studies have been conducted over the last several decades to investigate the immunological landscape of NPC using hematoxylin and eosin (H&E) staining, immunohistochemical (IHC) staining, and flow cytometry. Recent studies have demonstrated that the immunological components such as CD8+ T-cell infiltration and PD1/PDL1 expression may have prognostic value, but the results are still controversial (22–24). Asides from TILs composition, a few recent studies have shown that the spatial architecture of the TIME may also play an essential role in mediating cancer progression (25, 26). Thus, investigating the TIME composition and spatial architecture of NPC samples may provide additional critical insights into the complex and heterogeneous immunological landscape associated with disease progression.

The present study provides a comprehensive analysis of the composition and abundance of TILs, as well as PDL1 expression in the TME using the multiplex fluorescent immunohistochemistry (mfIHC) approach, aiming to evaluate the prognostic role of TILs in NPC. Furthermore, the spatial architecture of TCs and TILs is studied using multispectral imaging analysis. This allows researchers to assess the role of TILs’ intercellular proximity and distribution pattern in mediating disease progression, revealing a potential treatment-responsive biomarker for immune-modulatory therapy.

## Materials

### Study population

In this study, patients with NPC who were staged as I–IVA according to the 8^th^ American Joint Committee on Cancer (AJCC) TNM staging system, had no concomitant immune system disease, received IMRT at our institution between March 2010 and July 2014, and had sufficient tumor sample collection prior to any anticancer treatment were included. Eligible patients were then divided into two groups with comparable clinicopathological characteristics but distinct posttreatment outcomes. For attaining a balance between the two groups, clinicopathological data such as age, sex, smoking history, histological classification, AJCC 8^th^ TNM stage, Karnofsky performance status, lactate dehydrogenase level, hemoglobin level, platelet count, and treatment modality were considered. The 5-year disease progression rate was the main prognostic index in this study. Finally, 121 patients were included, with 68 in the favorable group surviving at least 5 years without disease progression (Group 1) and 53 in the unfavorable group having disease progression within 5 years (Group 2).

### Samples for mfIHC stains

All fresh tumor samples were preserved at our institution at −80°C liquid nitrogen with signed written informed consent. Formalin-fixed, paraffin-embedded (FFPE) blocks were prepared using a standard method. All H&E-stained slides were reassessed independently by two pathologists, and they were blinded to clinical data. After reviewing H&E-stained slides, one 1.5 mm diameter tumor tissue core from representative sections of FFPE blocks was used to construct the tissue microarray (TMA) (Shanghai Outdo Biotech Co., Ltd).

### Seven-color immunohistochemical multiplex

The Opal 7-color manual IHC kit 50 slides (Akoya, NEL811001KT) was used according to the manufacturer’s protocol. Briefly, TMA block sections were deparaffinized in an automatic dyeing machine (Leica ST5020, Leica) and subjected to antigen retrieval by microwave treatment in Citrate buffer (pH=6.0). Sections were then incubated in 3% hydrogen peroxide in methanol for 30 min at room temperature and subsequently with a blocking solution containing 0.3% bovine serum albumin in 0.05% Tween solution for 30 min. Then, the sections were incubated with primary antibody for 60 min at room temperature and its corresponding HRP-conjugated secondary antibody for 10 min, followed by opal fluorophores for 10 min. The staining sequence of primary antibodies and corresponding fluorescence channels was anti-CD4, CD3, PDL1, CD8, Foxp3 and CK, with the corresponding opal fluorophores 520, 690, 570, 620, 540 and 650, respectively. After staining the above markers in turn, sections were counterstained with 4′,6-diamidino-2-phenylindole (DAPI) (Life Tech) and mounted with VECTASHIELD fluorescence mounting medium (Vector Labs, Burlingame, CA).

### TILs phenotyping

To study the infiltration composition of T-cell subpopulations in NPC and their potential correlation with posttreatment progression, the following markers were used: cytokeratin (CK), CD3, CD4, CD8, Foxp3, and 4′,6-diamidino-2-phenylindole (DAPI,nuclear stain). CK was used to identify epithelial cancer cells in NPC tumor samples. The detailed information on biomarkers and antibodies used is presented in **Supplementary Table 1**. T-cells were identified using the CD3 marker. Furthermore, T-cell subpopulations were identified according to the standard staining protocol as cytotoxic T lymphocytes (CTLs, CD3+CD8+), CD4+ effector T-cells (Teffs, CD3+CD4+Foxp3-), Tregs (CD3+CD4+Foxp3+), and other T-cells (CD3+CD4-CD8-Foxp3-) (27). Finally, all other cells that were not recruited in our phenotyping categories, such as normal nasopharyngeal epithelial cells, blood vessels, nerves, macrophages, and other nuclear cells, were grouped into one category and labeled as “others.”

### Multispectral imaging

Multiplex fluorescent-stained TMA slides were scanned using the image analysis software StrataQuest (TissueGnostics-StrataQuest 7.7.1.165 version) for multicellular contextual tissue analysis in both bright field and fluorescence images. The total counts of various cell phenotypes derived from all available cores were analyzed. Additionally, the core density was calculated by dividing the total number of cells by the area of each core (cells/mm^2^). The spectral signature for each fluorophore was determined using single-antigen staining and captured using a multispectral fluorescent microscope, which records an image every 10 nm over the full-emission spectrum. This enabled the simultaneous capture of seven different fluorophores into a single composite image, which could then be unmixed and separated into six unique images representing each fluorophore and the nuclear stain DAPI, as well as the precise *x* and *y* spatial coordinates of each identified cell.

### PDL1 evaluation

PDL1 immunostaining was observed in the membrane and/or cytoplasm of the TCs and lymphocytes. At the cellular level, PDL1 expression was measured as the percentage of tumor or immune cells with positive staining (range: 0%–100%). At the patient level, specific phenotypes of cells with a PDL1 staining score of at least 5% were considered positive PDL1 expression (22). Since the evaluation and cutoff values for TILs are not standardized, the immune cell count in our study was based on a predetermined threshold of fluorescence intensity, which was identified by the mean value of fluorescence intensity of stained cells manually counted at a magnification (200×) in 10 random views. The median density of immune cells was chosen to divide the patient cohort into high and low expression groups (28). All staining was assessed by two independent pathologists who were blinded to the clinicopathologic data.

### Spatial distribution

The spatial distribution of TILs surrounding TCs was first analyzed in various tissue compartments (inner tumor vs. stroma). Next, the stroma area within 200 µm of the tumor edges was divided into 10 intervals, and the infiltration density of each type of TILs within each interval was quantitatively estimated. Furthermore, the Gcross function (G*ij*(r)) was calculated to estimate the distribution probability of finding at least one specified point “*j*” within a given radius “*r*” (µm) of any specified point “*i*,” allowing quantification of the relative proximity of any two cell types (29). Therefore, the Gcross function value becomes a quantitative index of TILs infiltration when the “*i*” is applied as a TC and “*j*” as TILs, with greater G_TC_ : _TIL_ values indicating a higher TILs infiltration density near TCs. Additionally, the area under the curve (AUC) of the Gcross curve was calculated to represent the accumulated infiltration level of cell type “*j*” within a given distance from cell type “*i*.” Accordingly, larger AUCs indicate higher immune cell interaction around TCs. Typical Gcross function curves indicating high (left), intermediate (middle), and low (right) levels of infiltration are illustrated in **Supplementary Figure 1**.

### Statistical analysis

The continuous variables, such as percentage, density, and Gcross value, were presented as median and interquartile range (IQR) and compared between two groups using the Mann–Whitney U-test. The categorical data were compared using the Chi-squared test. Disease-free survival (DFS) was defined as the time between the first date of diagnosis and disease progression or death, whereas overall survival (OS) was defined as the time between the first day of treatment and death from any cause or the last follow-up. The survival index was estimated using the Kaplan–Meier method, and the significance of the difference was assessed using the log-rank test. The Cox proportional hazards regression model was used to identify factors related to survival variables and to calculate the hazard ratio and corresponding confidence interval. To further evaluate the prognostic significance of TME, the densities and spatial architectures of TIL phenotypes were respectively assessed in multivariable Cox regression models that initially included age, sex, smoking history, histological type, N stage, T stage, and TNM stage. All statistical tests were two-sided, and a *p*-value of 0.05 or less was considered statistically significant. All statistical analyses were conducted by using the GraphPad Prism 8.0 software (GraphPad Software Inc.), the Statistical Package for the Social Sciences (SPSS) 22.0 software (IBM Inc.), and the R 3.6.3 software (R Foundation for Statistical Computing).

## Results

### Patients ‘ clinicopathological characteristics

In the final analysis, 121 patients with NPC were included and classified into two groups: favorable (Group 1, n = 68) and unfavorable (Group 2, n = 53). The overall population had a median follow-up time of 78.0 months (IQR: 57.5–93.2 months). The 5-year OS and DFS rates in the favorable and unfavorable groups were 100% vs. 45.3% (*p* < 0.001) and 100% vs. 0% (*p* < 0.001), respectively. **Table 1** demonstrates the clinicopathological characteristics of all studied patients. The median age in the favorable and unfavorable groups was 48 and 47 years, respectively, with males accounting for the majority of patients in both groups. Nearly 90% of patients had stage III to IVA diseases and received concurrent chemoradiotherapy. The pathological classification of all patients was nonkeratinizing undifferentiated subtype. Furthermore, the clinicopathological characteristics were comparable between the two groups.

**Table 1:** General characteristics of patients in two groups.

Characteristics	Group 1 (n=68)	Group 2 (n=53)	*P* value
	n (%)	n (%)	
Age (years)			0.964
Median(range)	48 (18-76)	47 (23-74)	
Sex			0.667
Male	53 (77.9)	43 (81.1)	
Female	15 (22.1)	10 (18.9)	
Smoking history			0.845
No	29 (43.3)	22 (41.5)	
Yes	38 (56.7)	31 (58.5)	
Histological type			0.900
WHO II	29 (42.6)	22 (41.5)	
WHO III	39 (57.4)	31 (58.5)	
T stage			0.900
T1	11 (16.2)	10 (18.9)	
T2	14 (20.6)	9 (17.0)	
T3	22 (32.4)	20 (37.7)	
T4	21 (30.9)	14 (26.4)	
N stage			0.082
N0	7 (10.3)	2 (3.8)	
N1	18 (26.5)	9 (17.0)	
N2	21 (30.9)	28 (52.8)	
N3	22 (32.4)	14 (26.4)	
Overall stage			0.653
I	2 (2.9)	1 (1.9)	
II	6 (8.8)	2 (3.8)	
III	23 (33.8)	22 (41.5)	
IVA	37 (54.4)	28 (52.8)	
KPS			1.000
<80	3 (4.4)	2 (3.8)	
≥80	65 (95.6)	51 (96.2)	
LDH(U/L)			0.085
<245	67 (98.5)	48 (90.6)	
≥245	1 (1.5)	5 (9.4)	
HB (g/L)			0.327
<130	9 (13.2)	11 (20.8)	
≥130	59 (86.8)	42 (79.2)	
PLT (×10^9^/L)			0.432
<300	59 (88.1)	44 (83.0)	
≥300	8 (11.9)	9 (17.0)	
Treatment pattern			0.825
CCRT	44 (64.7)	33 (62.2)	
IC+CCRT	10 (14.7)	10 (18.9)	
RT/IC+RT/CCRT+AC	14 (20.6)	10 (18.9)	

WHO II, non-keratinizing differentiated carcinoma; WHO III, non-keratinizing undifferentiated carcinoma; KPS, karnofsky performance status; LDH, lactate dehydrogenase; HB, haemoglobin; PLT, platelet; CCRT, concurrent chemoradiotherapy; IC, induction chemotherapy; RT, radiation therapy; AC, adjuvant chemotherapy.

### TILs subpopulation heterogeneity and disease progression

**Figure 1:**
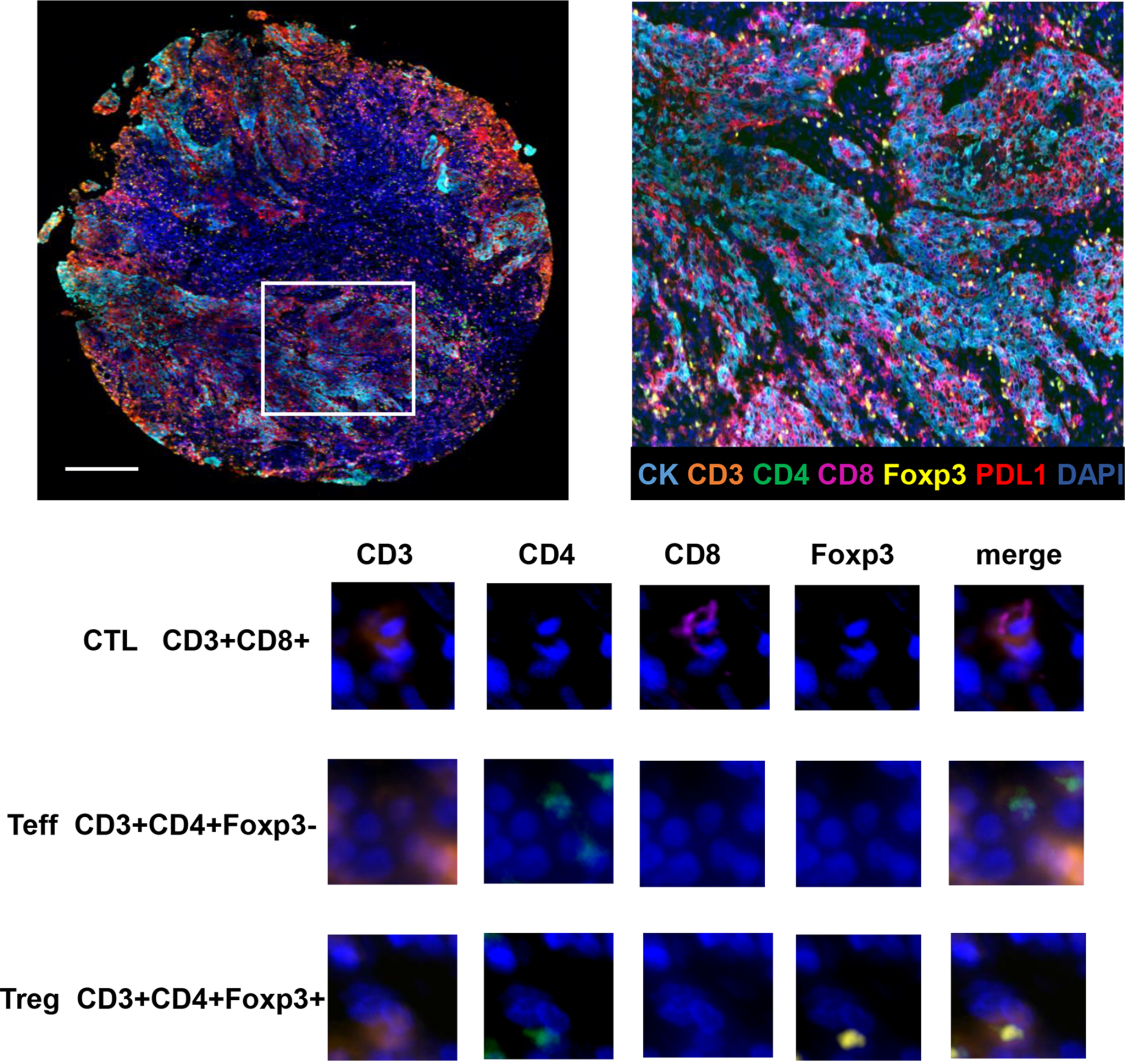
Opal seven-color multiplex analysis of NPC tumor tissue identifies specific TILs subtypes. Representative image of multiplex fluorescence staining and the enlarged subsection are displayed on the top panel. In the lower panel, images for unmixed single marker of CD3, CD4, CD8 and Foxp3 are presented in the left four columns. The right column demonstrates merged fluorescence image of various combination of four markers, resulting in the identification of typical TILs phenotypes such as CTLs, Teffs and Tregs.

**Figure 2:**
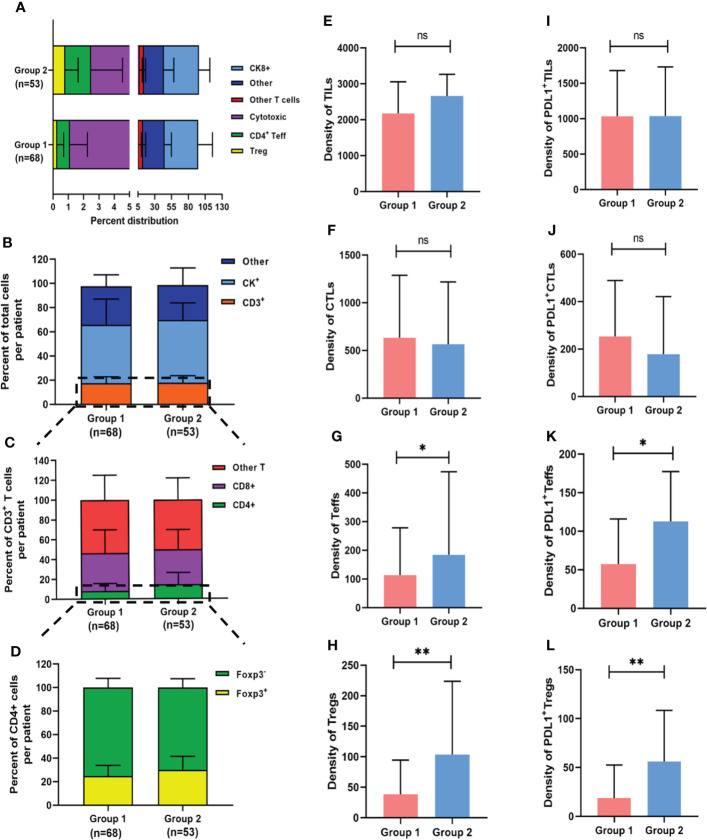
Composition of heterogeneous infiltrating immune cell subpopulations in Group 1 and Group 2. **(A)** Relative distribution of all analyzed cell phenotypes in NPC sample tissues. **(B-D)** Relative distribution analysis of different T cell subtypes between two groups, firstly by separating the total cell number into other, CK+ cells or CD3+ T cells (including all T cell subtypes) **(B)**; then focusing on CD3+ T cells and dividing them into CD4+ T (CD3+CD4+), CD8+ (CD3+CD8+) and other (CD3+CD4-CD8-) cells **(C)**; and finally focusing on CD3+CD4+ T cells and dividing them into Foxp3+ and Foxp3- T cells **(D)**. **(E-H)** Pairwise comparisons of the density of TIL subpopulations between the two groups for TILs **(E)**, CTLs **(F)**, Teffs **(G)** and Tregs **(H)**. **(I-L)** Pairwise comparisons of PDL1 positive TIL subpopulations between the two groups. *p < 0.05, **p < 0.01, ns, not significant.

TILs subpopulations and PDL1 expression on TCs and TILs were assessed for each core using mfIHC staining. **Figure 1** shows a representative immunofluorescence image. When the TILs compositions of the two groups were compared, patients in Group 2 had a significantly higher proportion of Tregs than those in Group 1 (0.8% vs. 0.3%, respectively, *p* = 0.023), whereas there was no significant difference in the proportions of TCs, total TILs, Teffs, and CTLs (**Figures 2A–D**).

Aside from cell proportion, cell density (calculated by dividing cell counts by area) can reflect cell distribution to some extent. The median densities of the total TILs, CTLs, Teffs, and Tregs were 2260.1 (IQR: 1721.1–3213.1), 582.1 (IQR: 242.7–1225.1), 140.1 (IQR: 69.8–318.6), and 58.2 (IQR: 19.0–142.0) cells/mm^2^, respectively. There were no significant differences in total TILs or CTLs density between the two groups (**Figures 2E, F**), but the median Tregs density (103.6 vs. 34.0 cells/mm^2^, *p* = 0.002) and median Teffs density (184.4 vs. 113.3 cells/mm^2^, *p* = 0.023) were significantly higher in patients with an unfavorable outcome than in those with a favorable outcome, respectively (**Figures 2G, H**). The detailed densities of each subtype are presented in **Supplementary Table 2**. When the median value was used as a cutoff, no significant associations were found between the infiltration densities of any of the TILs subpopulations and clinicopathological characteristics (**Supplementary Table 3**), implying that TILs have a prognostic impact that is independent of clinicopathological features.

Furthermore, PDL1 expression was assessed in various cell subtypes. The median percentages of PDL1 positive TCs (PDL1+ TCs) and PDL1 positive TILs (PDL1+ TILs) in the favorable and unfavorable groups were 20.3% vs. 18.6% (*p* = 0.775) and 71.1% vs. 60.0% (*p* = 0.166), respectively. In the overall population, the median densities of PDL1+ TCs and PDL1+ TILs were 3113.8 (IQR: 1043.4–5461.6) and 1037.4 (IQR: 711.5–1709.1) cells/mm^2^, respectively. In terms of PDL1 expression on TILs subpopulations, densities of PDL1+ Tregs (56.1 vs. 17.7 cells/mm^2^, *p* = 0.001) and PDL1+ Teffs (112.7 vs. 58.0 cells/mm^2^, *p* = 0.011) were significantly higher in patients with unfavorable outcomes than in those with favorable ones, respectively, with no significant differences in densities of PDL1+ TCs, PDL1+ TILs, or PDL1+ CTLs between the two groups (**Figures 2I–L**).

When the median value was used as a cutoff, no significant association was found between the infiltration densities of PDL1+ TCs, PDL1+ TILs, or any PDL1+ TILs subpopulations and the clinicopathological characteristics (**Supplementary Table 4**), implying that the higher infiltration density of PDL1+ Tregs and PDL1+ Teffs in the unfavorable group was independent of other prognostic factors for NPC.

### Tregs spatial distribution and disease progression

**Figure 3:**
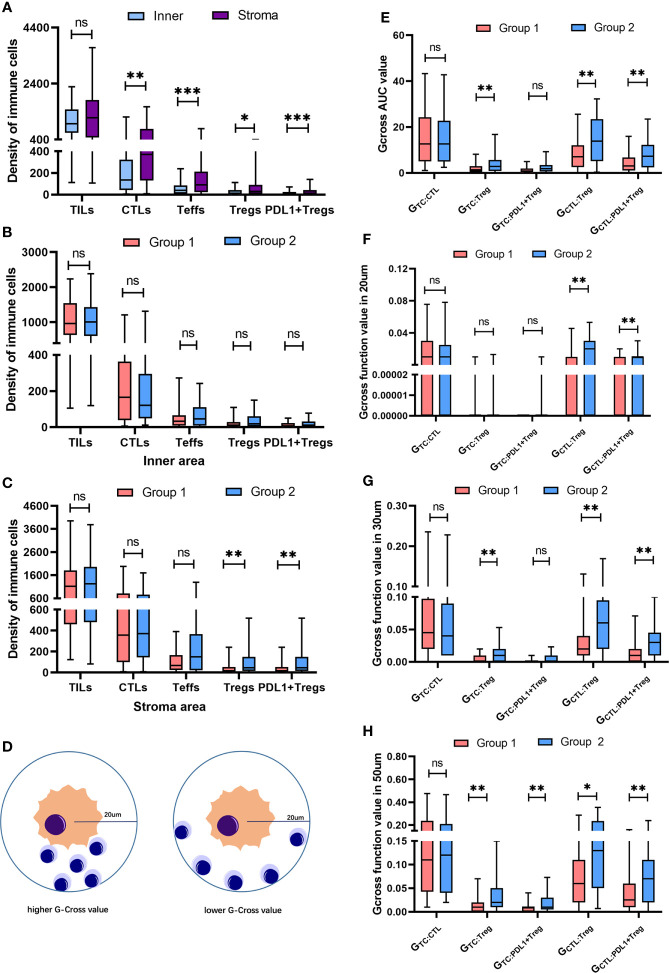
Spatial distribution of heterogeneous infiltrating immune cell subpopulations. **(A)** Pairwise comparison of the infiltration differences for TILs, CTLs, Teffs, Tregs and PDL1+ Tregs in the whole population between inner and stroma area. **(B)** Pairwise comparison of the infiltration differences for TILs, CTLs, Teffs, Tregs and PDL1+ Tregs within inner area between two groups. **(C)** Pairwise comparison of the infiltration differences for TILs, CTLs, Teffs, Tregs and PDL1+ Tregs in stroma area between two groups. **(D)** Schematic model for different scenarios of infiltration with the same number of immune cells locating within a 20 um radius of the tumor cell, reflecting distinct engagement level of immune cells. **(E)** Pairwise comparisons of the Gcross-AUC values for G_TC_ : _CTL_, G_TC_ : _Treg_, G_TC_ : _Teff_, G_TC_ : _PDL1+Treg_, G_CTL_ : _Treg_ and G_CTL_ : _PDL1+Treg_ between two groups. **(F-H)** Pairwise comparisons of the G-cross values at 20 um **(F)**, 30 um **(G)** and 50 um **(H)** radii for G_TC_ : _CTL_, G_TC_ : _Treg_, G_TC_ : _Teff_, G_TC_ : _PDL1+Treg_, G_CTL_ : _Treg_ and G_CTL_ : _PDL1+Treg_ between two groups. *p < 0.05, **p < 0.01, ***p < 0.001, ns, not significant.

The spatial distribution pattern of TILs subpopulations was analyzed to further investigate the impact of Tregs and PDL1+ Tregs on disease progression. Despite the similarity in the total TILs infiltration between the inner and stroma areas in the overall population, the stroma area had significantly higher infiltrations of CTLs (*p* = 0.001), Teffs (*p* < 0.001), Tregs (*p* = 0.04), and PDL1+ Tregs (*p* < 0.001) (**Figure 3A**). There was no significant difference between the two groups in terms of total TILs, CTLs, Teffs, Tregs, or PDL1+ Tregs engagement within the inner tumor area (**Figure 3B**). Patients in the unfavorable group had greater infiltration of Tregs (*p* = 0.008) and PDL1+ Tregs (*p* = 0.015) within the stroma area (**Figure 3C**). Detailed infiltration densities of TIL subpopulations in the inner and stromal areas are presented in **Supplementary Table 5**. The Gcross function was used as a more precise descriptive method to better investigate spatial intercellular interactions. **Figure 3D** depicts a schematic model for various scenarios of infiltration with the same number of immune cells located within a 20 µm radius of the TC. Despite the same infiltration density, the Gcross function can better reflect the distinct engagement level of immune cells. Within a 100 µm radius, the AUCs of the Gcross functions reflecting various cellular interactions were compared between the two groups, with patients with poor outcomes having significantly higher Gcross AUCs of the TC : Tregs (**Figure 3E**). **Figures 3F–H** show Gcross function values at specific radii of 20, 30, and 50 µm. Similarly, patients with poor outcomes had significantly higher G_TC_ : _Treg_ at radii of 30 and 50 µm, as well as higher G_TC: PDL1+ Treg_ at the radius of 50 µm. Detailed Gcross function values for each radius for the two groups are presented in **Supplementary Table 6**. The spatial distributions of Tregs and PDL1+ Tregs surrounding CTLs were also investigated, in addition to intercellular distances between TILs and TCs. The Gcross AUCs of CTL : Treg and CTL : PDL1+ Treg were significantly higher in the unfavorable group, implying that Tregs and PDL1+ Tregs are strongly engaged in the proximity of CTLs. Consistent with the AUC analysis, both G_CTL_ : _Treg_ and G_CTL: PDL1+ Treg_ at the specific radius of 20, 30, and 50 µm were significantly higher in patients with disease progression, indicating the potential role of intercellular interaction between Tregs, PDL1+ Tregs, and CTLs in mediating tumor progression. It should be noted that both Tregs and PDL1+ Tregs had much higher infiltration probabilities near CTLs than TCs (**Supplementary Table 7**).

### Univariate and multivariate analyses for OS and DFS

In the overall study population, **Table 2** presents univariate and multivariate analyses of density and Gcross function score of TILs subpopulations for OS and DFS. Patients with higher densities of Tregs, PDL1+ Teffs, and PDL1+ Tregs had significantly lower DFS in univariate analysis. Patients with more abundant PDL1+ Tregs infiltration had a lower OS, with a *p*-value approaching statistical significance. Further multivariate analysis revealed that higher infiltrations of Tregs, Teffs, PDL1+ Teffs, and PDL1+ Tregs were significantly associated with lower DFS, whereas only abundant PDL1+ Tregs infiltration may be associated with lower OS trending toward significance.

**Table 2:** Univariate and multivariate analysis for OS and DFS according to densities and Gcross functions of TILs subpopulations.

Variables	Median IQR (cells/mm^2^)	Disease-free survival		Overall survival	
		Univariate HR (95% CI)	Multivariate HR (95% CI)^a^	Univariate HR (95% CI)	Multivariate HR (95% CI)^a^
Density					
TILs	2260.1 (1721.2-3213.1)
High vs Low		1.13 (0.66-1.94)	1.27 (0.73-2.22)	1.12 (0.56-2.22)	1.19 (0.58-2.42)
*p* value		0.647	0.402	0.747	0.636
CTLs	582.1 (242.7-1225.1)				
High vs Low		0.84 (0.49-1.44)	0.75 (0.42-1.32)	0.77 (0.38-1.53)	0.69 (0.34-1.42)
*p* value		0.539	0.317	0.45	0.316
Teffs	140.1 (69.8-318.6)				
High vs Low		1.64 (0.94-2.84)	1.83 (1.03-3.23)	1.47 (0.73-2.95)	1.38 (0.67-2.82)
*p* value		0.078	0.039	0.279	0.382
Tregs	58.2 (19.0-142.0)				
High vs Low		1.79 (1.03-3.12)	1.94 (1.09-3.42)	1.71 (0.85-3.46)	1.71 (-.83-3.51)
*p* value		0.040	0.023	0.136	0.146
PDL1+TILs	1037.4 (711.5-1709.1)				
High vs Low		0.90 (0.53-1.55)	0.934 (0.54-1.62)	1.01 (0.54-2.13)	1.23 (0.61-2.46)
*p* value		0.701	0.807	0.836	0.563
PDL1+TCs	3113.8 (1043.4-5461.6)				
High vs Low		0.78 (0.46-1.34)	0.76 (0.42-1.35)	1.39 (0.70-2.77)	0.72 (0.35-1.47)
*p* value		0.371	0.351	0.342	0.369
PDL1+CTLs	223.7 (76.0-466.6)				
High vs Low		0.74 (0.43-1.27)	0.68 (0.38-1.20)	0.54 (0.26-1.09)	0.55 (0.26-1.13)
*p* value		0.274	0.180	0.085	0.105
PDL1+Teffs	69.7 (33.5-148.3)				
High vs Low		1.87 (1.07-3.27)	2.07 (1.18-3.65)	1.27 (0.64-2.52)	1.40 (0.69-2.83)
*p* value		0.027	0.012	0.497	0.348
PDL1+Tregs	32.2 (11.5-63.7)				
High vs Low		2.34 (1.32-4.14)	2.54 (1.42-4.56)	2.00 (0.98-4.08)	2.01 (0.98-4.15)
*p* value		0.003	0.002	0.055	0.058
Gcross function value^b^					
G_TC_ : _Treg_	1.80 (0.55-4.35)				
High vs Low		2.35 (1.33-4.15)	2.43 (1.34-4.42)	2.27 (1.10-4.69)	2.20 (1.04-4.63)
*p* value		0.003	0.004	0.026	0.039
G_TC_ : _PDL1+Treg_	1.07 (0.40-2.61)				
High vs Low		1.61(0.93-2.79)	1.68 (0.96-2.95)	1.62 (0.81-3.26)	1.57 (0.77-3.18)
*p* value		0.090	0.072	0.176	0.215
G_CTL_ : _Treg_	9.19 (3.49-17.12)				
High vs Low		2.01 (1.14-3.52)	2.16 (1.21-3.87)	1.96 (0.96-4.00)	1.78 (0.86-3.68)
*p* value		0.015	0.009	0.066	0.121
G_CTL_ : _PDL1+Treg_	4.21 (1.38-9.41)				
High vs Low		2.08 (1.18-3.65)	2.15 (1.21-3.80)	2.03 (0.99-4.15)	2.08 (1.00-4.29)
*p* value		0.011	0.009	0.053	0.049
20 um radius					
G_TC_ : _Treg_	0.0008 (0.0002-0.0031)				
High vs Low		1.67 (0.96-2.89)	1.75 (0.98-3.12)	1.42 (0.71-2.83)	1.44 (0.70-2.95)
*p* value		0.069	0.060	0.32	0.326
G_TC_ : _PDL1+Treg_	0.0006 (0.0000-0.0017)				
High vs Low		1.21 (0.71-2.07)	1.23 (0.70-2.15)	1.09 (0.55-2.15)	1.04 (0.51-2.10)
*p* value		0.493	0.467	0.808	0.918
G_CTL_ : _Treg_	0.0089 (0.0014-0.0212)				
High vs Low		2.29 (1.29-4.04)	2.39 (1.33-4.29)	2.31 (1.11-4.80)	2.06 (0.99-4.30)
*p* value		0.004	0.003	0.025	0.053
G_CTL_ : _PDL1+Treg_	0.0034 (0.000-0.0104)				
High vs Low		2.01 (1.15-3.53)	2.14 (1.21-3.80)	1.70 (0.84-3.44)	1.79 (0.88-3.64)
*p* value		0.011	0.009	0.139	0.110
30 um radius					
G_TC_ : _Treg_	0.0037 (0.001-0.0111)				
High vs Low		2.17 (1.24-3.81)	2.25 (1.24-4.09)	1.98 (0.98-4.04)	2.01 (0.97-4.19)
*p* value		0.007	0.008	0.058	0.062
G_TC_ : _PDL1+Treg_	0.0026 (0.0006-0.0067)				
High vs Low		1.64 (0.94-2.84)	1.72 (0.97-3.04)	1.63 (0.81-3.28)	1.62 (0.79-3.22)
*p* value		0.080	0.065	0.170	0.183
G_CTL_ : _Treg_	0.0326 (0.0088-0.0670)				
High vs Low		2.26 (1.28-3.40)	2.37 (1.31-4.30)	2.29 (1.20-4.75)	1.91 (0.91-4.01)
*p* value		0.005	0.004	0.027	0.087
G_CTL_ : _PDL1+Treg_	0.0161 (0.0028-0.0319)				
High vs Low		2.20 (1.25-3.87)	2.43 (1.37-4.30)	2.14 (1.04-4.37)	2.20 (1.07-4.54)
*p* value		0.006	0.002	0.038	0.032
50 um radius					
G_TC_ : _Treg_	0.0127 (0.0036-0.0342)				
High vs Low		2.42 (1.37-4.28)	2.38 (1.32-4.30)	2.33 (1.13-4.80)	2.19 (1.05-4.58)
*p* value		0.002	0.004	0.022	0.037
G_TC_ : _PDL1+Treg_	0.0071 (0.0027-0.0201)				
High vs Low		1.99 (1.12-3.49)	2.00(1.12-3.56)	2.19 (1.06-4.51)	2.07 (0.99-4.32)
*p* value		0.017	0.019	0.034	0.054
G_CTL_ : _Treg_	0.0842 (0.0265-0.1597)				
High vs Low		1.78 (1.02-3.10)	1.91 (1.07-3.41)	1.70 (0.84-3.44)	1.56 (0.76-3.21)
*p* value		0.042	0.028	0.139	0.230
G_CTL_ : _PDL1+Treg_	0.0384 (0.0110-0.0855)				
High vs Low		2.34 (1.32-4.14)	2.45 (1.38-4.38)	2.37 (1.14-4.91)	2.35 (1.12-4.92)
*p* value		0.003	0.003	0.021	0.023

a ,Cox proportional hazards regression model adjusted for age, sex, N stage, T stage, TNM stage, smoking history, histological type.

b ,Gcross function values measured as the probability of finding at least one Treg/PDL1+Tregs within a given radius from a tumor cell or CTLs.

CI, confidence interval; HR, hazard ratio; IQR, interquartile range.

TILs, tumor infiltrating lymphocytes; CTLs, cytotoxic T lymphocytes; Teffs, positive effector T cells; Tregs, regulatory T cells; PDL1+ TILs, PDL1 positive tumor infiltrating lymphocytes; PDL1+TCs, PDL1 positive tumor cells; PDL1+CTLs, PDL1 positive cytotoxic T lymphocytes; PDL1+Teffs, PDL1 positive effector T cells; PDL1+Tregs, PDL1 positive regulatory T cells.

**Figure 4:**
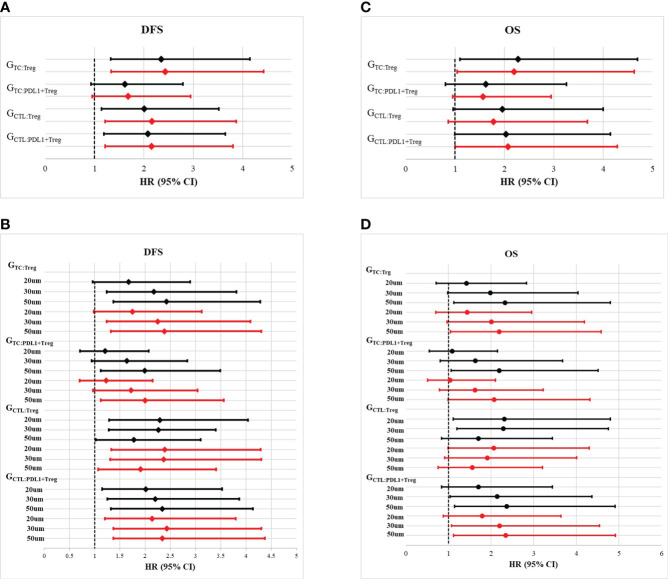
Forest plots of multivariate analysis and univariate analysis of Gcross function score in the whole cohort. **(A)** Hazard ratio of univariate (black solid line) and multivariate (red solid line) analysis of G_TC_ : _Treg_, G_TC_ : _PDL1+Treg_, G_CTL_ : _Treg_ and G_CTL_ : _PDL1+Treg_ for disease free survival (DFS). **(B)** Hazard ratio of univariate (black solid line) and multivariate (red solid line) analysis of G_TC_ : _Treg_, G_TC_ : _PDL1+Treg_, G_CTL_ : _Treg_ and G_CTL_ : _PDL1+Treg_ within certain raidus (20um, 30um and 50um) for disease free survival (DFS). **(C)** Hazard ratio of univariate (black solid line) and multivariate (red solid line) analysis of G_TC_ : _Treg_, G_TC_ : _PDL1+Treg_, G_CTL_ : _Treg_ and G_CTL_ : _PDL1+Treg_ for overall survival (OS). **(D)** Hazard ratio of univariate (black solid line) and multivariate (red solid line) analysis of G_TC_ : _Treg_, G_TC_ : _PDL1+Treg_, G_CTL_ : _Treg_ and G_CTL_ : _PDL1+Treg_ within certain raidus (20um, 30um and 50um) for overall survival (OS).

Furthermore, the impact of Tregs spatial architecture on disease progression was investigated. Univariate analysis of the Gcross function revealed that the TC : Treg, TC : PDL1+ Treg, CTL : Treg, and CTL : PDL1+ Treg colocalizations were all associated with worse DFS to varying degrees. Further multivariate analysis confirmed that higher G_TC_ : _Treg_, G_TC_ : _PDL1+ Treg_, G_CTL_ : _Treg_, and G_CTL_ : _PDL1+ Treg_ all had independently negative effects on DFS. **Table 2** and **Figures 4A, B** present detailed data on the univariable and multivariable analyses for DFS.

Although TC : Treg, TC : PDL1+ Treg, CT : Treg, and CTL : PDL1+ Treg colocalization within a certain radius had a significant correlation with OS, multivariable analysis revealed that only G_TC_ : _Treg_ and G_CTL_ : _PDL1+ Treg_ were independently correlated with lower OS. **Table 2** and **Figures 4C, D** present detailed data on univariate and multivariate Cox regression analyses for OS.

**Figure 5:**
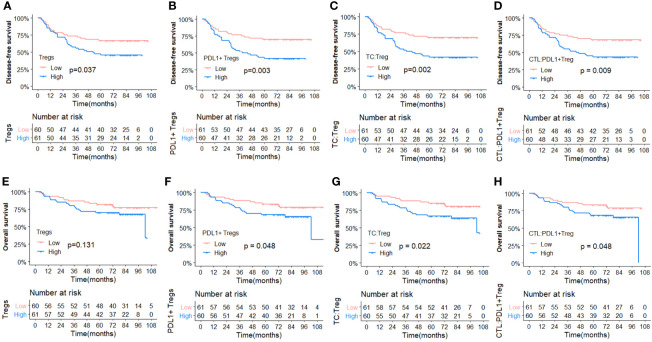
Comparisons of Kaplan-Meier survival curves between high and low infiltration density of TILs and Gcross function scores. (Upper panel) Disease free survival curves for Tregs **(A)**, PDL1+ Tregs **(B)**, G_TC_ : _Treg_ **(C)** and G_CTL_ : _PDL1+Treg_ **(D)**. (Lower panel) Overall survival curves for Tregs **(E)**, PDL1+ Tregs **(F)**, G_TC_ : _Treg_ **(G)** and G_CTL_ : _PDL1+Treg_ **(H)**.

To better investigate the prognostic role of the aforementioned elements, **Figure 5** depicts the Kaplan–Meier survival curves for OS and DFS between subgroups with high vs. low density of Tregs, PDL1+ Tregs, and the high vs. low Gcross functions of TC : Treg and CTL : PDL1+ Treg. The survival curves for OS and DFS between subgroups with high and low infiltration, as well as other TIL colocalization, are shown in **Supplementary Figure 2**.

## Discussion

In this study, we comprehensively analyzed the composition and spatial distribution of TILs and PDL1 expression in NPC using mfIHC and multispectral imaging analysis. Tregs and PDL1+ Tregs compositionally higher density and spatial closeness to TCs were significantly associated with worse outcomes. Furthermore, increased Tregs engagement, particularly PDL1+ Tregs surrounding CTLs, was highly associated with poor outcomes. Overall, our findings demonstrate that a suppressive immune microenvironment had a propelling effect on NPC progression regardless of potential clinical confounders, with crucial implications for prognosis prediction and immune-modulatory therapy.

TILs are well known for their vital role in mediating antitumor immune responses. Cellular factors including myeloid-derived suppressor cells (MDSCs), tumor-associated macrophages(TAMs), CD8+ T cells and regulatory T cells (Tregs) in TME also may impact the prognosis of sloid tumors (30). Therefore, a thorough understanding of the diversity and complexity of TME emerges as a crucial approach to identifying more valid biomarkers for failure prediction and therapeutic targets. Previous studies on the prognostic significance of TILs in various tumors, including NPC, have yielded conflicting results. Wang et al. (19) and Almangush et al. (23) used H&E-stained slides to assess the prognostic value of TILs in endemic and nonendemic areas of NPC, respectively. Both studies found that overall TILs were significantly associated with survival, whereas TILs subtypes were not further evaluated. Ooft et al. (24), Al-Rajhi et al. (31), and Zhu et al. (32) found that increasing intratumoral CD3+ TILs infiltration was associated with superior OS and DFS without further investigation of subphenotypes. Ono et al. investigated TILs subpopulations and found that higher CTLs density was a significant factor in favorable prognosis (33). However, in our study, neither the abundance nor the density of TILs or CTLs was found to be associated with clinical outcomes. In agreement with our finding, Larbcharoensub et al. found that CTLs abundance was not associated with a significant difference in clinical survival (34). These inconsistencies suggest the presence of significant heterogeneity in TME and the need for further investigation of TME’s impact on the antitumor immune response.

Tregs play crucial roles in suppressing antitumor immunity in TME by expressing ligands for inhibitory checkpoint receptors and secreting suppressive cytokines, promoting the occurrence and development of tumors (35, 36). Therefore, it is not surprising that Tregs are often associated with a poor prognosis in cancer. Although the high density of Foxp3 positive TILs was consistently associated with poor survival in patients with operable tongue cancer (37), breast cancer (38), hepatocellular cancer (39), ovarian cancer (40), and esophageal cancer (41), it was also reported to be associated with favorable outcomes in patients with head and neck squamous cell cancer (42), colorectal cancer (43), and SCLC (44). Such controversial findings have also been reported in patients with NPC. Ooft et al. found that a high Foxp3 count was an independent predictor of better OS (45). In our study, patients with a higher infiltration of Tregs had a significantly inferior OS and DFS, which was consistent with Lu’s study findings (46). Lab work conducted by Huo et al. demonstrated that EBV-EBNA1 enhanced the chemotactic migration of Treg cells through the TGFβ1-SMAD3-PI3K-AKT-c-JUN-miR-200a-CXCL12-CXCR4 axis in NPC microenvironment, thereby promoting NPC immune escape (47). Alternatively, Tregs can secrete immunosuppressive cytokines including TGF-β, IL-10, and IL-35, and subsequently suppress cytotoxic effect of CD8 positive CTL and effector T cells (Teff) (48, 49). Therefore, overcoming the suppressive signal of Tregs may be critical to restoring exhausted CTLs function and enhancing patient responsiveness to immune-modulatory therapy.

The PD1/PDL1 axis is a well-known immune checkpoint that attenuates T-cells’ antitumor immune response and mediates immunological escape (50). Despite the fact that a considerable number of studies assessed the prognostic value of PDL1 expression in NPC, the results were inconsistent among studies (23, 24, 32, 33, 51–55). Zhang et al. and Li et al. reported that high PDL1 expression on TCs was significantly associated with poor DFS or OS (55) (56). However, Zhu et al. found that positive PDL1 expression on TCs is a favorable prognostic factor in patients with NPC (32). Conversely, Liu et al. found that high PDL1 expression on TILs and TCs was highly associated with decreased local recurrence in patients with NPC after radiotherapy (54). Similarly, Ono et al. demonstrated that patients with higher PDL1 expression on TILs had longer progression-free survival and OS (33). However, another two previous studies found no association between PDL1 expression on TILs and survival outcomes (34, 51). Likewise, neither PDL1+ TCs nor PDL1+ TILs densities were found to be associated with survival outcomes in patients with NPC in our present study.

Aside from PDL1 expression on TCs and overall TILs, our study used the mfIHC method to conduct a more extensive and meticulous investigation into the prognostic significance of PDL1 expression on TILs subphenotypes. The combination of mfIHC, high-quality image acquisition, and multispectral imaging analysis, as advanced technology, allows for simultaneous multimarker labeling as well as cellular proximity analysis in a single core of tissue, providing a novel insight into TME research. One notable finding in the present study was that patients with more abundant PDL1+ Treg infiltration had the worst survival. A similar scenario has been reported in other solid tumors. DiDomenico et al. found that PDL1 was important in the expansion and maintenance of Tregs immunosuppression activity in glioma (57). Furthermore, Wu et al. found that the frequency of PD-L1^hi^ Tregs was positively correlated with PD-1-positive CD8 in the tumor stroma of non-small cell lung cancer (58). Additionally, Wu et al. found that PD-1^hi^ CD8 with PD-L1^hi^ Tregs group had the lowest proportion of tumor necrosis factor-alpha- and interferon-gamma-producing CTLs while achieving the best response to PD-1 blockade immunotherapy. Based on the present research, it is plausible to speculate that PDL1 inhibitors may aid in the recovery of CTL tumor-killing capacity by attenuating PDL1+ Treg suppression, thereby introducing another appealing mechanism of PD1/PDL1 axis blockade. De et al. reported that tumor infiltrating Tregs can express surface specific molecules such as PD-L1 and PD-L2 in order to bind their receptors on the surface of CD8+ T cells, inhibiting CD8+ T-cell activation, which also supported our outcomes and hypothesis (36).

In addition to the composition of the TILs subpopulation, our study revealed the intercellular spatial association in TME. Although a few studies have researched the TME either by using mIF (59) or by applying spatial analysis (60) in NPC, mIF based TME composition and spatial structure have not been comprehensively investigated. As far as we know, our study was the first to investigate both the compositional abundance and the spatial distribution of TILs in NPC. In the present study, Gcross analysis was adopted to quantify the intercellular proximity between any two types of cells. Herein, radii of 20, 30, 50, and 100 µm were selected as distances of interest for this study since distances between 20 and 110 µm have been previously suggested to represent physiological distances for direct intercellular crosstalk (25, 61). According to Gcross analysis, significant engagement of Tregs surrounding TCs was independently associated with poor outcomes. These findings are consistent with recent findings in lung cancer (15) and esophageal cancer (17), highlighting the significance of the close proximity of Tregs to TCs in prompting progression.

Another significant finding of the present study is that closer and denser infiltration of PDL1+ Tregs surrounding CTLs was independently associated with a worse outcome. Furthermore, we found that Tregs and PDL1+ Tregs had a substantially higher probability of infiltrating near CTLs than TCs. These findings support our hypothesis that PDL1+ Tregs interact with CTLs *via* the PD1/PDL1 axis and subsequently mediate CTL dysfunction in antitumor activity, resulting in enhanced immune suppression. This has implications for future clinical investigations and mechanisms of prognosis prediction, as well as immunotherapy for patients with NPC.

Our study also has other strengths. To the best of our knowledge, this is the first study to investigate both the compositional abundance and the spatial distribution of TILs in NPC. Second, our study population included two groups of patients with well-matched characteristics but distinct DFS, which reduced the confounding effect of traditional prognostic factors and aided the identification of valid differential immunomarkers. Third, rather than semiquantitative measurements, the mfIHC technology allows for the codetection of multiple markers at a single cell level, demonstrating the high quality of cell phenotyping and accurate cell densities.

Nevertheless, there are several limitations to our study. First, patients in our study received treatment in the early 2010s, when EBV DNA was not well known as a prognostic factor. Therefore, the EBV DNA data in our database were incomplete and thus were not considered in the present study. Second, we only investigated classical TILs subpopulations along with PDL1 expression, whereas other markers of TILs functional state were not covered in this study. Future studies incorporating alternative lymphocyte markers could offer a more comprehensive landscape of TME. Third, although we established the prognostic role of PDL1+ Tregs infiltration in NPC, other immune-suppressive cell populations, such as MDSCs and M2 macrophages, may also play vital roles in immune suppression. Future studies should delve into more abundant cell subpopulations to provide a more precise cell–cell interaction network. Finally, TMA cannot represent the whole slide, just as the whole slide cannot represent the whole tumor. The heterogeneity always exists within the tumor, especially in those with large tumor burden. However, many studies have shown good concordance rate between TMA and whole slide (62).

To conclude, our study comprehensively demonstrates the infiltrating profile and spatial distribution characteristics of TILs in NPC. Increased Tregs infiltration, particularly PDL1+ Tregs, as well as their proximity to TCs and CTLs, correlates with unfavorable outcomes, highlighting the essential role of dynamic intercellular interactions between heterogeneous T-cell subtypes in disease progression. This study offers new insights into the immunological landscape of NPC, adding evidence of the prognostic value of TILs and the potential mechanism of PDL1/PD1 axis blockade in the era of immune-modulatory therapy.

## Data availability statement

The data used and/or analyzed during the current study are available from the corresponding author on reasonable request.

## Ethics statement

The study was approved by the Institutional Review Board (IRB) of Cancer Hospital, Chinese Academy of Medical Sciences and Peking Union Medical College (IRB approval no. NCC 2462). All patients provided informed consent for the collection of tissue samples.

## Author contributions

FZ and GS: data curation, formal analysis, methodology, visualization, writing-original draft preparation. XC, YZ, RW, JZ, XH, KW, and YQ: investigation, data curation. SS, QL, YL, and XS: data curation, methodology. JL and Y-XL: investigation, supervision. BL and JW: software, visualization. JY and JW: conceptualization, project administration, writing - review and editing, and funding acquisition. All authors contributed to the article and approved the submitted version.

## Funding

This work was supported by the CAMS Innovation Fund for Medical Sciences (2021-I2M-C&T-B-070) to JW, the Beijing Hope Run Special Fund of Cancer Foundation of China (LC2021L06) and the National Natural Science Foundation of China grant (81172125) to JY.

## Acknowledgments

We would like to thank Dr. Chuqing Pan for her guidance with the spatial analysis.

## Conflict of interest

The authors declare that the research was conducted in the absence of any commercial or financial relationships that could be construed as a potential conflict of interest.

## Publisher’s note

All claims expressed in this article are solely those of the authors and do not necessarily represent those of their affiliated organizations, or those of the publisher, the editors and the reviewers. Any product that may be evaluated in this article, or claim that may be made by its manufacturer, is not guaranteed or endorsed by the publisher.

## Abbreviations

NPC, nasopharyngeal carcinoma; PDL1, programmed cell death-ligand 1; PD1, programmed cell death protein 1; TILs, tumor infiltrating lymphocytes; OS, overall survival; DFS, disease free survival; CTLs, cytotoxic T lymphocytes; Teffs, effector T cells; Tregs, regulatory T cells; IC, immune cell; TC, tumor cell; IQR, interquartile range; HR, hazard ratio; CI, confidence interval; KPS, Karnofsky performance status.
