# Supplementary material for: Spatial architecture of regulatory T-cells correlates with disease progression in patients with nasopharyngeal cancer
Source: Front Immunol. 2022 Nov 10;13:1015283. doi: 10.3389/fimmu.2022.1015283 (PMC9684321; doi:10.3389/fimmu.2022.1015283)
Supplement: Supplementary file 1 [file DataSheet_1.docx]

**Supplementary Table 1. Sequential Opal multiplex staining protocol.**

| Antigen | Primary antibody | | Catalogue number | TSA fluorophore |
| --- | --- | --- | --- | --- |
|  | Concentration | Provider |  |  |
| Pan-CK | 1:1.5 | Abcarta | PA125 | Opal 650 |
| CD3 | 1:10 | DAKO | GA503 | Opal 690 |
| CD4 | Ready to use | Abcarta | PA285 | Opal 520 |
| CD8 | 1:500 | Abcarta | 85338 | Opal 620 |
| Foxp3 | 1:1000 | Abcam | ab4728 | Opal 540 |
| PD-L1 | Ready to use | Genetech | GT2280 | Opal 570 |

**Supplementary Table 2. Percentage and density of TILs phenotypes.**

| Variables | Group 1 | Group 2 | *p* value |
| --- | --- | --- | --- |
|  | Median (IQR) | Median (IQR) |  |
| **Percentage (%)** | | | |
| TCs | 51.5 (36.6-69.4) | 50.5(34.2-71.3) | 0.775 |
| CTLs | 5.1 (1.9-9.3) | 4.9 (2.0-8.6) | 0.936 |
| Teffs | 0.8 (0.4-2.0) | 1.7 (0.6-3.8) | 0.122 |
| Tregs | 0.3 (0.1-0.7) | 0.8 (0.20-1.6) | 0.023 |
| Other cells | 32.2(14.4-42.0) | 29.8 (15.2-44.3) | 0.775 |
| Other T cells | 6.8 (4.3-11.0) | 6.5 (4.1-8.5) | 0.514 |
| **Density (cells/mm^2^)** | | | |
| TILs | 2230.1(1724.4-3193.0) | 2431.8 (1645.2-3217.7) | 0.612 |
| CTLs | 632.6 (239.7-1303.9) | 564.8 (244.5-1159.8) | 0.908 |
| Teffs | 113.3 (57.1-278. 7) | 184.4 (80.6-474.0) | 0.023 |
| Tregs | 34.0 (13.5-94.4) | 103.6 (26.5-223.7) | 0.002 |
| PDL1+TILs | 1049.8 (706.5-1727.0) | 1036.4 (703.9-1665.6) | 0.839 |
| PDL1+CTLs | 253.5(71.0-515.8) | 178.5 (91.0-407.9) | 0.707 |
| PDL1+Teffs | 58.0 (17.7-116.0) | 112.7 (40.8-177.4) | 0.011 |
| PDL1+Tregs | 17.7 (6.1-52.5) | 56.1 (16.7-108.4) | 0.001 |
| PDL1+TCs | 3342.2 (1171.2-6221.2) | 2324.9 (991.9-5204.0) | 0.352 |
| Abbreviations: IQR, interquartile range; TCs, tumor cells; TILs, tumor infiltrating T lymphocytes; CTLs, cytotoxic T lymphocytes; Teffs, CD4 positive effector T cells; Tregs, regulatory T cells; PDL1+ TILs, PDL1 positive tumor infiltrating lymphocytes; PDL1+TCs, PDL1 positive tumor cells; PDL1+CTLs, PDL1 positive cytotoxic T lymphocytes; PDL1+Teffs, PDL1 positive effector T cells; PDL1+Tregs, PDL1 positive regulatory T cells. | | | |

**Supplementary Table 3. Association of TILs density with clinicopathological characteristics**

| Variables | TILs | | | Teffs | | | CTLs | | | Tregs | | |
| --- | --- | --- | --- | --- | --- | --- | --- | --- | --- | --- | --- | --- |
|  | low | high | *p* | low | high | *p* | low | high | *p* | low | high | *p* |
| Age (years) |  |  | 0.854 |  |  | 0.888 |  |  | 0.770 |  |  | 0.415 |
| ≤48 | 36 (60.0) | 35 (57.4) |  | 35 (59.3) | 36 (58.1) |  | 35 (57.4) | 36 (60.0) |  | 33 (55.0) | 38 (62.3) |  |
| ＞48 | 24 (40.0) | 26 (42.6) |  | 24 (40.7) | 26 (41.9) |  | 26 (42.6) | 24 (40.0) |  | 27 (45.0) | 23 (37.7) |  |
| Sex |  |  | 0.178 |  |  | 0.593 |  |  | 0.171 |  |  | 0.786 |
| Male | 51 (85.0) | 45 (73.8) |  | 48 (81.4) | 48 (77.4) |  | 53 (84.1) | 46 (74.2) |  | 47 (78.3) | 49 (80.3) |  |
| Female | 9 (15.0) | 16 (26.2) |  | 11 (18.6) | 14 (22.6) |  | 10 (15.9) | 16 (25.8) |  | 13 (21.7) | 12 (19.7) |  |
| Smoking |  |  | 0.712 |  |  | 0.677 |  |  | 0.915 |  |  | 0.318 |
| No | 24 (40.0) | 27 (45.0) |  | 26 (44.1) | 25 (40.3) |  | 26 (42.6) | 25 (41.7) |  | 28 (46.7) | 23 (37.7) |  |
| Yes | 36 (60.0) | 33 (55.0) |  | 33 (55.9) | 37 (59.7) |  | 35 (57.4) | 35 (58.3) |  | 32 (53.3) | 38 (62.3) |  |
| KPS |  |  | 1.000 |  |  | 0.689 |  |  | 0.634 |  |  | 0.680 |
| <80 | 2 (3.3) | 3 (4.9) |  | 2 (3.4) | 3 (4.8) |  | 2 (3.3) | 3 (5.0) |  | 3 (5.0) | 2 (3.3) |  |
| ≥80 | 58 (96.7) | 58 (95.1) |  | 57 (96.6) | 59 (95.2) |  | 59 (96.7) | 57 (95.0) |  | 57 (95.0) | 59 (96.7) |  |
| T stage |  |  | 0.346 |  |  | 0.559 |  |  | 0.114 |  |  | 0.655 |
| T1/T2 | 19 (31.7) | 25 (41.0) |  | 23 (39.0) | 21 (33.9) |  | 18 (29.5) | 26 (43.3) |  | 23 (38.3) | 21 (34.4) |  |
| T3/T4 | 41 (68.3) | 36 (59.0) |  | 36 (61.0) | 41 (66.1) |  | 43 (70.5) | 34 (56.7) |  | 37 (61.7) | 40 (65.6) |  |
| N stage |  |  | 0.843 |  |  | 0.826 |  |  | 0.126 |  |  | 0.694 |
| N0/N1 | 17 (28.3) | 19 (31.1) |  | 17 (28.8) | 19 (30.6) |  | 22 (36.1) | 14 (23.3) |  | 19 (31.7) | 17 (27.9) |  |
| N2/N3 | 43 (71.7) | 42 (68.9) |  | 42 (71.2) | 43 (69.4) |  | 39 (63.9) | 46 (76.7) |  | 41 (68.3) | 44 (72.1) |  |
| TNM |  |  | 1.000 |  |  | 0.301 |  |  | 0.358 |  |  | 0.107 |
| I/II | 8 (8.3) | 6 (9.8) |  | 7 (11.9) | 4 (6.5) |  | 7(11.5) | 4(6.7) |  | 8 (13.3) | 3 (4.9) |  |
| III/IV | 55 (91.7) | 55 (90.2) |  | 52 (88.1) | 58 (93.5) |  | 54 (88.5) | 56 (93.3) |  | 52 (86.7) | 58 (95.1) |  |
| Pathology |  |  | 0.200 |  |  | 0.677 |  |  | 0.144 |  |  | 0.318 |
| WHO II | 29 (48.3) | 22 (36.1) |  | 26 (44.1) | 25 (40.3) |  | 30 (49.2) | 21 (35.0) |  | 28 (46.7) | 23 (37.7) |  |
| WHO III | 31 (51.7) | 39 (63.9) |  | 33 (55.9) | 37 (59.7) |  | 31 (50.8) | 39 (65.0) |  | 32 (53.3) | 38 (62.3) |  |

Abbreviations: TILs, tumor infiltrating T lymphocytes; CTLs, cytotoxic T lymphocytes; Teffs, effector T cells; Tregs, regulatory T cells; WHO II, non-keratinizing differentiated carcinoma; WHO III, non-keratinizing undifferentiated carcinoma; KPS, karnofsky performance status.

**Supplementary Table 4. Association of PDL1+ TCs and PDL1+TILs density with clinicopathological characteristics**

| Variables | PDL1+TCs | | | PDL1+TILs | | | PDL1+ CTLs | | | PDL1+ Teffs | | | PDL1+ Tregs | | |
| --- | --- | --- | --- | --- | --- | --- | --- | --- | --- | --- | --- | --- | --- | --- | --- |
|  | low | high | *p* | low | high | *p* | low | high | *p* | low | high | *p* | low | high | *p* |
| Age (years) |  |  | 0.379 |  |  | 0.656 |  |  | 0.142 |  |  | 0.854 |  |  | 0.581 |
| ≤48 | 37 (62.7) | 34(54.8) |  | 34  (56.7) | 37  (60.7) |  | 31  (51.7) | 40  (65.6) |  | 35  (57.4) | 36  (60.0) |  | 34  (55.7) | 37  (61.7) |  |
| ＞48 | 22(37.3) | 28(45.2) |  | 26(43.3) | 24(39.3) |  | 29(48.3) | 21(34.4) |  | 26(42.6) | 24(40.0) |  | 27(44.3) | 23(38.3) |  |
| Sex |  |  | 0.932 |  |  | 0.786 |  |  | 1.000 |  |  | 1.000 |  |  | 0.654 |
| Male | 47(79.7) | 49(79.0) |  | 47  (78.3) | 49  (80.3) |  | 48  (80.0) | 48  (78.7) |  | 48  (78.7) | 48  (80.0) |  | 47  (77.0) | 49  (81.7) |  |
| Female | 12 (20.3) | 13 (21.0) |  | 13  (21.7) | 12  (19.7) |  | 12  (20.0) | 13  (21.3) |  | 13  (21.3) | 12  (20.0) |  | 14  (23.0) | 11  (18.3) |  |
| Smoking |  |  | 0.291 |  |  | 0.603 |  |  | 1.000 |  |  | 1.000 |  |  | 0.715 |
| No | 22 (37.3) | 29 (46.8) |  | 26 (41.3) | 28 (45.9) |  | 25 (41.7) | 26 (43.3) |  | 26 (42.6) | 25 (42.4) |  | 27 (44.3) | 24  (40.7) |  |
| Yes | 37 (62.7) | 35 (53.2) |  | 37 (58.7) | 33 (54.1) |  | 35 (58.3) | 34 (56.7) |  | 37 (57.4) | 34 (57.6) |  | 34 (55.7) | 35  (59.3) |  |
| KPS |  |  | 1.000 |  |  | 0.661 |  |  | 1.000 |  |  | 0.680 |  |  | 0.680 |
| <80 | 2 (3.4) | 3 (4.8) |  | 2 (3.3) | 3 (4.9) |  | 2 (3.3) | 3 (4.9) |  | 2 (3.3) | 3 (5.0) |  | 2 (3.3) | 3 (5.0) |  |
| ≥80 | 57 (96.6) | 59 (95.2) |  | 58 (96.7) | 58 (95.1) |  | 58 (96.7) | 58 (95.1) |  | 58 (96.7) | 57 (95.0) |  | 59 (96.7) | 57 (95.0) |  |
| T stage |  |  | 0.837 |  |  | 0.149 |  |  | 0.089 |  |  | 0.708 |  |  | 0.851 |
| T1/T2 | 22 (37.3) | 22 (35.5) |  | 18 (30.0) | 26 (42.6) |  | 17 (27.9) | 27 (44.3) |  | 21 (34.4) | 23 (38.3) |  | 23 (37.7) | 21 (35.0) |  |
| T3/T4 | 37 (62.7) | 40 (64.5) |  | 42 (70.0) | 35 (57.4) |  | 43 (71.7) | 34 (55.7) |  | 40 (65.6) | 37 (61.7) |  | 38 (62.3) | 39 (65.0) |  |
| N stage |  |  | 0.330 |  |  | 0.953 |  |  | 0.164 |  |  | 0.694 |  |  | 0.843 |
| N0/N1 | 20 (33.9) | 16 (25.8) |  | 18 (30.0) | 18 (29.5) |  | 22 (36.1) | 14 (23.3) |  | 17 (27.9) | 19 (31.7) |  | 19 (31.1) | 17 (28.3) |  |
| N2/N3 | 39 (66.1) | 46 (74.2) |  | 42 (70.0) | 43 (70.5) |  | 39 (63.9) | 46 (76.7) |  | 44 (72.1) | 41 (68.3) |  | 42 (68.9) | 43 (71.7) |  |
| TNM |  |  | 0.388 |  |  | 0.358 |  |  | 0.762 |  |  | 0.762 |  |  | 0.529 |
| I/II | 4 (6.8) | 7 (11.3) |  | 4 (6.7) | 7 (11.5) |  | 6 (10.0) | 5 (8.2) |  | 5 (8.2) | 6 (10.0) |  | 7 (11.5) | 4 (6.7) |  |
| III/IV | 55 (93.2) | 55 (88.7) |  | 56 (93.3) | 54 (88.5) |  | 54 (90.0) | 56 (91.8) |  | 56 (91.8) | 54 (90.0) |  | 54 (88.5) | 56 (93.3) |  |
| Pathology |  |  | 0.677 |  |  | 0.794 |  |  | 1.000 |  |  | 0.583 |  |  | 1.000 |
| WHO II | 26 (44.1) | 25 (40.3) |  | 26 (43.3) | 25 (41.0) |  | 25 (41.7) | 26 (42.6) |  | 24 (39.3) | 27 (45.0) |  | 26 (42.6) | 25 (41.7) |  |
| WHO III | 33 (55.9) | 37 (59.7) |  | 34 (56.7) | 36 (59.0) |  | 35 (58.3) | 35 (57.4) |  | 37 (60.7) | 33 (55.0) |  | 35 (57.4) | 35 (58.3) |  |

Abbreviations: PDL1+TCs, PDL1 positive tumor cells;PDL1+TILs, PDL1 positive tumor infiltrating lymphocytes; PDL1+CTLs, PDL1 positive cytotoxic T lymphocytes; PDL1+Teffs, PDL1 positive effector T cells; PDL1+Tregs, PDL1 positive regulatory T cells; WHO II, non-keratinizing differentiated carcinoma; WHO III, non-keratinizing undifferentiated carcinoma; KPS, karnofsky performance status.

**Supplementary Table 5. Infiltration densities of TILs subpopulations in inner and stroma area**

| Density(cells/mm^2^) | Overall | | *p* | Inner | | *p* | Stroma | | *p* |
| --- | --- | --- | --- | --- | --- | --- | --- | --- | --- |
|  | Inner | Stroma |  | Group 1 | Group 2 |  | Group 1 | Group 2 |  |
| TILs | 964.3 | 1182.1 | 0.123 | 958.9 | 1008.2 | 0.936 | 1114.2 | 1233.2 | 0.655 |
| CTLs | 135.9 | 371.5 | 0.001 | 165.9 | 121.3 | 0.166 | 357.5 | 371.4 | 0.936 |
| Teffs | 38.7 | 90.7 | <0.001 | 33.1 | 46.6 | 0.416 | 66.8 | 148.8 | 0.122 |
| Tregs | 12.2 | 28.9 | 0.04 | 10.9 | 18.4 | 0.416 | 16.7 | 45.2 | 0.008 |
| PDL1+Tregs | 8.2 | 28.9 | <0.001 | 7.4 | 11.0 | 0.416 | 8.1 | 23.1 | 0.023 |

Abbreviations: TILs, infiltrating T lymphocytes; CTLs, cytotoxic T lymphocytes; Teff, effector T cells; Tregs, regulatory T cells; PDL1+ Tregs, PDL1 positive regulatory T cells.

**Supplementary Table 6. Gcross function value in two groups**

| **Gcross** | Group 1 | Group 2 |
| --- | --- | --- |
|  | Median(IQR) | Median(IQR) |
| **G_TC:CTL_** |  |  |
| AUC | 12.6064 (5.0284-24.2897) | 12.6688 (4.9666-22.7113) |
| 20 um | 0.0139 (0.0044-0.0331) | 0.0135 (0.0042-0.0260) |
| 30 um | 0.0450 (0.0164-0.0954) | 0.0443 (0.0144-0.0896) |
| 50 um | 0.1113 (0.0434-0.2367) | 0.1153 (0.0408-0.2140) |
| **G_TC:Treg_** |  |  |
| AUC | 1.3505 (0.4917-2.9091) | 2.8638 (0.8699-5.6255) |
| 20 um | 0.0007 (0.0001-0.0020) | 0.0016 (0.0003-0.0040) |
| 30 um | 0.0024 (0.0007-0.0073) | 0.0063 (0.0019-0.0163) |
| 50 um | 0.0083 (0.0028-0.0229) | 0.0250 (0.0068-0.0469) |
| **G_TC:PDL1+Treg_** |  |  |
| AUC | 0.8180 (0.2308-1.9786) | 1.8031 (0.6411-3.4159) |
| 20 um | 0.0003 (0.0000-0.0012) | 0.0007 (0.0001-0.0022) |
| 30 um | 0.0019 (0.0001-0.0048) | 0.0041 (0.0009-0.0098) |
| 50 um | 0.0059 (0.0012-0.0145) | 0.0133 (0.0047-0.0283) |
| **G_CTL:Treg_** |  |  |
| AUC | 7.0581 (2.6026-12.0359) | 13.8666 (5.1792-23.4609) |
| 20 um | 0.0048 (0.0000-0.0149) | 0.0180 (0.0030-0.0310) |
| 30 um | 0.0215 (0.0064-0.0432) | 0.0575 (0.0184-0.0957) |
| 50 um | 0.0584 (0.0209-0.1113) | 0.1306 (0.0513-2.349) |
| **G_CTL:PDL1+Treg_** |  |  |
| AUC | 3.0723 (1.0761-6.7970) | 7.2899 (2.4192-12.2266) |
| 20 um | 0.0008 (0.0000-0.0059) | 0.0007 (0.0011-0.0141) |
| 30 um | 0.0007 (0.0015-0.0211) | 0.0252 (0.0067-0.0440) |
| 50 um | 0.0235 (0.0078-0.0625) | 0.0701 (0.0209-0.1123) |

Abbreviation: IQR, interquartile range. TCs, tumor cells; CTLs, cytotoxic T cells; Tregs, regulatory T cells; PDL1+ Tregs, PDL1 positive regulatory T cells.

**Supplementary Table 7.** **The infiltration probabilities of Tregs and PDL1+Tregs to TCs and CTLs in entire group**

| **Gcross** | Median(IQR) | *p* value |
| --- | --- | --- |
| G_TC:Treg_ | 1.8008 (0.5525-4.3521) | <0.001 |
| G_CTL:Treg_ | 9.1268 (3.3719-16.3398) |  |
| G_TC:PDL1+Treg_ | 1.0714 (0.3967-2.6114) | <0.001 |
| G_CTL:PDL1+Treg_ | 4.2158 (1.3846-9.4125) |  |
| **20 um** |  |  |
| G_TC:Treg_ | 0.0008 (0.0002-0.0031) | <0.001 |
| G_CTL:Treg_ | 0.0089 (0.0014-0.0212) |  |
| G_TC:PDL1+Treg_ | 0.0006 (0.0000-0.0017) | 0.001 |
| G_CTL:PDL1+Treg_ | 0.0034 (0.0000-0.0104) |  |
| **30 um** |  |  |
| G_TC:Treg_ | 0.0037 (0.0010-0.0111) | <0.001 |
| G_CTL:Treg_ | 0.0326 (0.088-0.0670) |  |
| G_TC:PDL1+Treg_ | 0.0026 (0.0006-0.067) | <0.001 |
| G_CTL:PDL1+Treg_ | 0.0161 (0.0028-0.0319) |  |
| **50 um** |  |  |
| G_TC:Treg_ | 0.0127 (0.0036-0.0342) | <0.001 |
| G_CTL:Treg_ | 0.0842 (0.0265-0.1597) |  |
| G_TC:PDL1+Treg_ | 0.0064 (0.0022-0.0161) | <0.001 |
| G_CTL:PDL1+Treg_ | 0.0384 (0.0110-0.0855) |  |

Abbreviations: IQR, interquartile range; TCs, tumor cells; CTLs, cytotoxic T cells; Tregs, regulatory T cells; PDL1+ Tregs, PDL1 positive regulatory T cells.


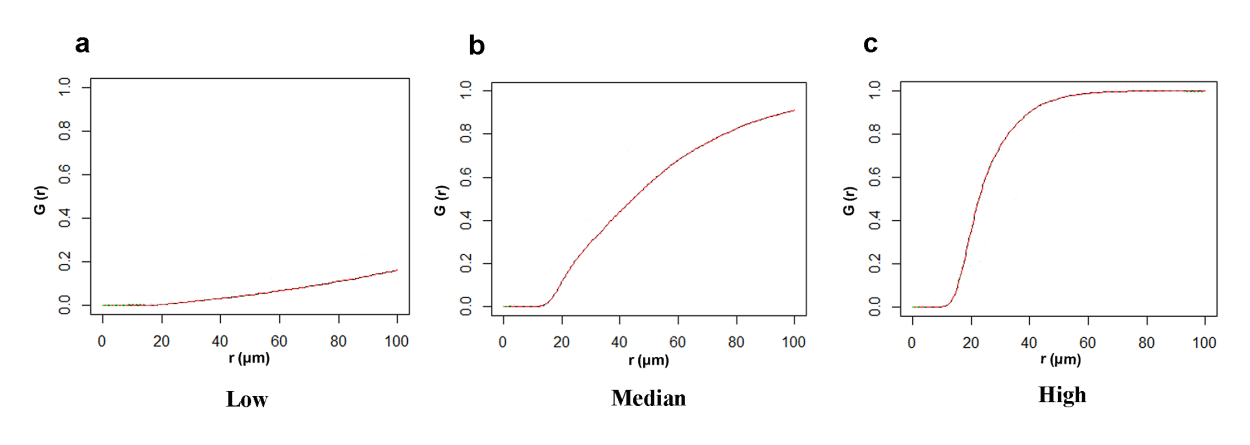


**Supplementary figure 1:** Typical G-cross function curves indicating high (a), intermediate (b) and low (c) levels of infiltration.


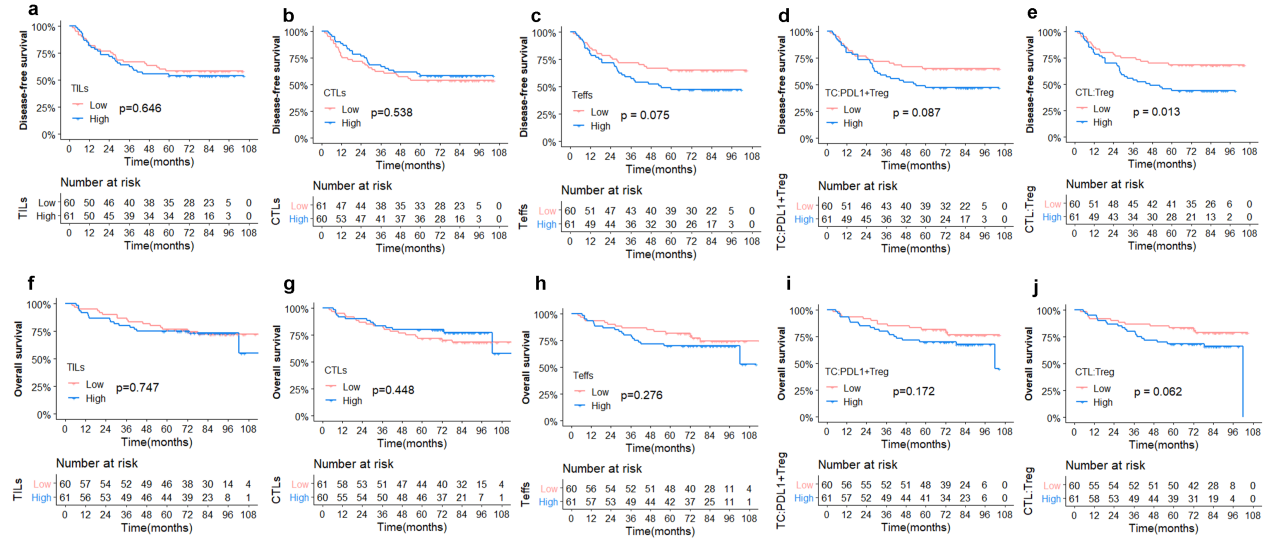


**Supplementary figure 2:** Disease free survival curves for TILs (a), CTLs (b), Teffs (c), G_TC:PD-L1+Treg_ (d), and G_CTL:Treg_ (e); overall survival curves for TILs (f), CTLs (g), Teffs (h),G_TC:PD-L1+Treg_ (i), and G_CTL:Treg_ (j).

TILs, tumor infiltrating T lymphocytes; CTLs, cytotoxic T cells; Teffs, effector T cells; Tregs, regulatory T cells; PDL1+ Tregs, PDL1 positive regulatory T cells.
